# Supplementary material for: Immediate and short-term effects of eccentric muscle contractions on structural, morphological, mechanical, functional and physiological properties of peripheral nerves: A protocol for a systematic review and meta-analysis
Source: PLoS One. 2023 Jul 28;18(7):e0289422. doi: 10.1371/journal.pone.0289422 (PMC10381088; doi:10.1371/journal.pone.0289422)
Supplement: Attachment — Submitted filename: Reviewer Comments to the manuscript PONE-D-22-32928.docx [file pone.0289422.s002.docx]

**Reviewer Comments to the manuscript** **PONE-D-22-32928, “Immediate and short-term effects of eccentric muscle contractions on structural, morphological, mechanical, functional and physiological properties of peripheral nerves: a protocol for a systematic review and meta-analysis”**

The aim of the study is to summarize in a systematic review the effects of eccentric exercise on the on structural, morphological, mechanical, functional and physiological properties of peripheral nerves. The study is well designed and the methods proposed are appropriate to answer the research question.

I have few comments and questions that aim to ameliorate the proposed study protocol.

**Introduction**

The rationale brought by the authors sustain the necessity of conduction this systematic review, however I would be interesting to be more clear about what it represents to muscle function and force production disruption of peripheral nerves following eccentric contraction. Does it relate to the observed decrease in force due to exercise induced muscle damage?

For disruption of peripheral nerves to occur, is it necessary to perform specific eccentric contraction (like isokinetic contractions) or does it also occur when performing functional daily living movements or strength exercises?

I might have missed it, but are these alteration transient? How many days (or hours) does it last? When observed, is the person more susceptible to injury?

As described by the authors, it seems that the peripheral nerve alterations observed after eccentric exercise are somewhat similar those of exercise induced muscle damage. If this is the case, are both things liked? And if so, I would suggest to bring it up.

These comments are made thinking that this is a topic that is not widely discussed so many readers might be reading it for the first time. Addressing possible functional alterations as well as wider possibilities to induce peripheral disruption might strengthen the rationale to and the necessity to study this topic.

**2.1.1 Type of studies and 2.1.2 Types of participants**

Does the inclusion of studies “evaluating the impact of eccentric contractions (applied to upper or lower limb muscles) on peripheral nerves dysfunction…”, isn’t it possible that the inclusion of individual with peripheral nerves dysfunction will interfere in the results when compared to health tissues? Also, in the following topic “2.1.2 Types of participants” it is specified that will be included studies with “healthy asymptomatic individuals”, isn’t it contradictory? I might be missing a point here.

Why are the following outcomes included:? **4. Physiology:** intraneural blood flow measured with Doppler US (B-mode with Colour Doppler)… alterations in signal intensity (T1 and T2 via MRI) and echo intensity (via high-resolution US)

To describe how the strength of the body of evidence, do the authors plane to use the Grading of Recommendations Assessment, Development and Evaluation (GRADE)?
